# Temperature-Assisted Gas-phase Silanization Using Different Silanes for Actomyosin-Based Nanodevices

**DOI:** 10.1021/acsomega.5c09878

**Published:** 2026-01-30

**Authors:** Tim Erichlandwehr, Jeremy P. Teuber, Rukan H. Nasri, Cagla Selalmaz, Marko Usaj, Alf Månsson, Irene Fernandez-Cuesta

**Affiliations:** † Institut für Nanostruktur- und Festkörperphysik, Universität Hamburg, Luruper Chausseee 149, 22761 Hamburg, Germany; ‡ Deutsches Elektronen-Synchrotron (DESY), Notkestraße 85, 22607 Hamburg, Germany; § Max Planck Institute for the Structure and Dynamics of Matter, Luruper Chaussee 149, 22761 Hamburg, Germany; ∥ Hamburg Centre for Ultrafast Imaging, Notkestraße 85, 22607 Hamburg, Germany; ⊥ Department of Chemistry and Biomedical Sciences, Linnaeus University, Norra Vagen 49, 39182 Kalmar, Sweden

## Abstract

Motor proteins drive motion in living systems. Myosin motors adsorbed on a surface propel actin filaments by hydrolyzing ATP. This makes them interesting systems for applications in nanotechnology, e.g. as sensors, for transporting molecular cargo or driving other forms of molecular motion. However, their effective functioning requires the proper combination of materials with adequate surface chemistry and hydrophobic properties. Here, we investigate a set of materials systems used as substrates and analyze their compatibility with the actomyosin system. As a reference, we used glass slides coated with trimethylchlorosilane (TMCS) where coating is performed in liquid phase, since this is a commonly used approach. We then explored an alternative vapor phase deposition method to coat glass slides with various silane compounds: in addition to TMCS, we also used perfluoro-octyltrichlorosilane (FOTCS) and perfluoro-dodecyltrichlorosilane (FDDTCS). In vitro motility assays (IVMAs), where surface-adsorbed myosin motor fragments propel actin filaments, were then used to measure the sliding velocity on the different surfaces. Filaments propelled on FOTCS-functionalized surfaces by chemical vapor deposition exhibited the highest average sliding velocity (3.9 ± 1.2 μm/s; mean ± SD) and retained a high fraction of motile actin filaments (87%), comparable to TMCS-functionalized surfaces (3.3 ± 0.4 μm/s, 90% motile). In addition, we also used a UV-curable polymer as active substrate material, which we have successfully treated to either promote or inhibit motor adsorption and therefore motility. We have evaluated the hydrophobic characteristics and the roughness of the different functionalized surfaces. In addition, we patterned microchannels with physical and chemical contrast, to confine the motor adsorption and consequently motion of the myosin- driven actin filaments to the patterned microchannel bottoms. This gas-phase deposition technique uses just a low cost commercial oven and offers a promising method for tailoring the surface properties of various materials, paving the way for standardizing and advancing the application of myosin-propelled actin filaments in nanotechnology and microdevices.

## Graphical abstract

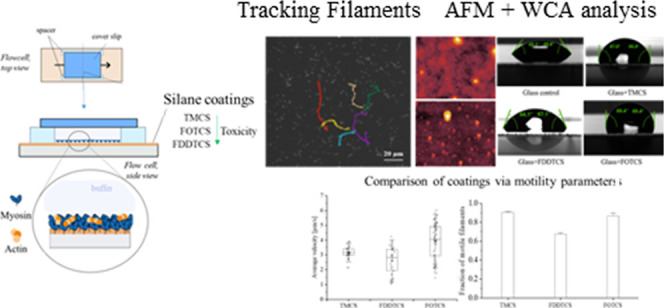

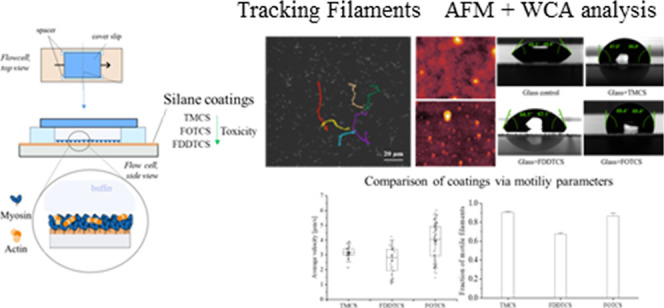

## Introduction

Motor proteins drive most of the nondiffusive movement in living organisms and produce forces for a range of purposes. They are responsible for muscle contraction as well as motion and force production by other cells. They also underlie active and directed transport, including the transport of organelles or biomolecules at the intracellular level. In addition to their fundamental role in biology, they can also be exploited as active elements in micro- and nanodevices, where they can pave the way for novel functionalities. Due to their low energy consumption and small size, motor proteins, which are driven by adenosine-5′-triphosphate (ATP), could replace pumps or other bulky devices that are conventionally used for active flow control.^1–3^ In addition, they find applications in biosensors, and sorting devices or for targeted drug delivery.^1,6,8,20,21,33,35^ Recently they have also been used in biocomputation to solve complex mathematical problems by searching through mazes.^3,4^

One motor protein that is widely used is myosin II from fast skeletal muscle tissue. Primarily, the myosin II subfragment, heavy meromyosin (HMM), obtained by enzymatic cleavage of full-length myosin, has been used to propel filamentous actin (F-actin). A key advantage of myosin and actin compared to alternative motor systems relying on kinesin and microtubules is the order of magnitude faster speed.^1,5–8^

The mechanism of HMM adsorption on a substrate is complex and not entirely understood yet, but the surface hydrophobicity is an essential parameter during this process.^2,5,9,10,34,36^ The HMM motor fragment consists of a hydrophobic tail and a more stable and more hydrophilic, actin-binding head with a predominantly positive electrostatic surface.^11^ Therefore, a hydrophobic surface will result in HMM adsorbing to the surface via its tail, with a free head moiety in the opposite direction, enabling actin binding. On the other hand, a hydrophilic surface with excess negative charge is expected to result in the head of the motors sticking to the surface, thereby losing its ability to bind and propel filaments. For this, one of the most extended approaches is to use substrates coated with nitrocellulose or with other polymers with hydrophobic properties.^7,16^ Another common method to control the surface hydrophobicity and significantly reduce surface charge is surface functionalization with silanes. Trimethylchlorosilane (TMCS) is typically used to coat glass or silicon surfaces by immersion dipping in liquid.^2,12,13^ The conversion of the hydroxyl groups on the glass surface into trimethylsilyl ethers imparts a hydrophobic character to the material, with surface water contact angles (WCA) in the range of 50–90°, enabling high motility of the actin-myosin systems, previously found to be optimal at angles of 70–80°.^10,11^

In the in vitro motility assay (IVMA), the motor proteins adsorb on a surface via their tail domain and propel their associated cytoskeletal filaments with a free head domain on top allowing quantification of the average sliding velocity of the motile filaments using light microscopy. In addition to its use in fundamental studies, the IVMA is also the basis for several of the applications based on motor proteins, e.g. by using them to transport cargoes along nanofabricated channels in nanoseparation, for biosensing or to compute the solution of combinatorial mathematical problems in network-based biocomputation.^2,5^ For the applications, one of the goals of the community working with such systems is to develop methods to control and guide the movement of the (actin) filaments to enable local, guided or directional transport. This can be achieved e.g., through physical walls that confine the filaments on their tracks,^3,10,12^ and/or through a chemical contrast of tracks with motility-promoting surfaces surrounded by nonpromoting surfaces.^1,5,6^

The fabrication of surfaces and/or micro and nanostructures and devices for the mentioned purposes should ideally be easy, scalable, and compatible with multiscale patterning, integrating features in the micro and nanoscale within the same device. Having fully transparent devices (as opposed to e.g. Silicon) could also be an additional advantage, to simplify the read-out during experiments. In addition, the chemicals involved should be as safe as possible, following the guidelines on replacing toxic chemicals for their safer alternatives.^14^ However, before the motors can be used in nanodevices, it is important to test and characterize the different materials involved in substrate and device fabrication. This is necessary to check the proper adhesion of the motors, and to quantify the resulting velocity of the propelled filaments for different substrate patterning conditions.

Here, we propose material systems and protocols improved (e.g., compared to Albet-Torres et al.^13^ and Lindberg et al.^10^) to achieve the above-mentioned goals for the fabrication of substrates for actin-myosin in vitro motility assays (IVMAs) and a silanization protocol to control the hydrophobicity of the surfaces. The method is based on gas phase deposition at high temperatures (70–90 °C), which is fast, requires low amounts of chemicals and further enables the coating with a variety of silane compounds, including those with long, aliphatic carbon chains. For this, we use a low cost, commercial, standard vacuum oven, where the temperature and the pressure can be reliably controlled, placing a vial with the silanes inside together with the sample to be functionalized. We used this method to coat glass substrates with TMCS, which is often used by the community,^10,12^ and with two other silanes, which are less hazardous. In all the cases we performed actin-myosin IVMAs, and compared the results to those obtained on glass substrates coated with TMCS using the standard, dip coating method. We have also used the vapor-phase deposition method to coat a flat polymer substrate (Ormostamp), which could be potentially used to make micro and nanostructured substrates via UV nanoimprinting. We have also made microchannels with chemical contrast, having hydrophilic polymer (Ormostamp) walls and hydrophobic glass bottom. This approach showed great selectivity with respect to motor immobilization and highlights the versatility of such a polymer, which can be used either for promoting or inhibiting motor adhesion and activity. Overall, the gas-phase deposition proved to be a suitable method to make substrates for IVMA with a variety of materials. The quality of the substrates is reliable and very reproducible, and, since the method relies on use of a commercial oven, it could be easy to transfer and implement in other laboratories, helping toward standardization.

## Experimental Section

### Silane Compounds

We used three different silanes: trimethylchlorosilane (TMCS), perfluoro-octyltrichlorosilane (FOTCS), and perfluoro-dodecyltrichlorosilane (FDDTCS), all purchased from Sigma-Aldrich Chemie GmbH. Figure 1 summarizes some of their physical and chemical properties, relevant to this work and the Globally Harmonized System of Classification and Labeling of Chemicals (GHS) symbols. These properties were obtained from the technical datasheets of the provider.

**Figure 1:**
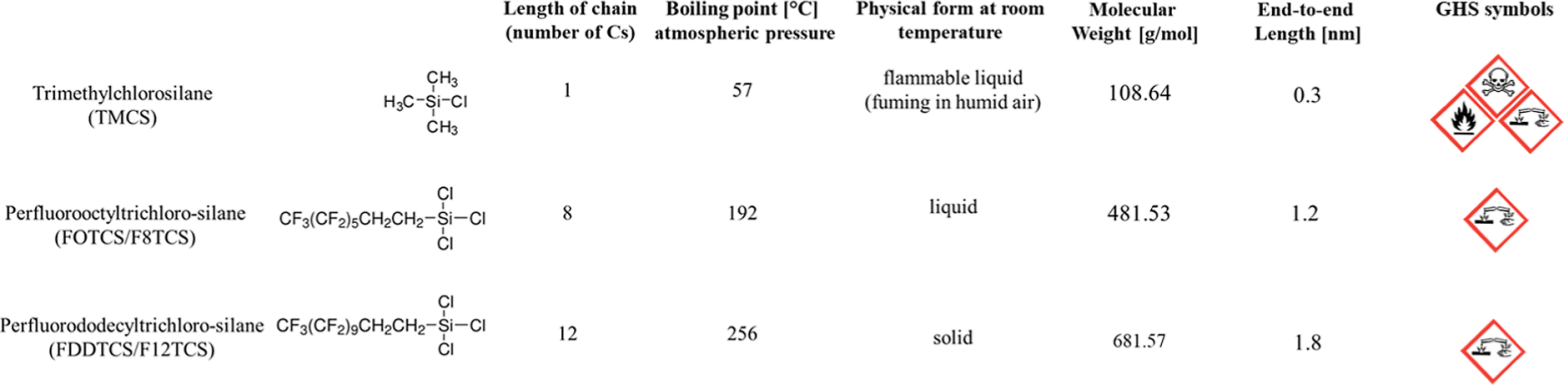
Silane compounds used for temperature assisted gas-phase silanization: name, molecular composition, chain length (number of carbon atoms), boiling point at atmospheric pressure, appearance at room temperature, molecular weight, length and GHS symbols. Information obtained from the provider and the material safety datasheets (MSDS). More information on hazards in Table S2 in Supporting Information.

### Temperature Assisted Gas-phase Silanization

To control the surface hydrophobicity, glass and polymer samples were coated with silanes in gas phase in a vacuum oven. To this end, the sample surface was first activated by UV-Ozone (144AX-220 model, Jelight Co Inc.) for 2.5 min. The effectiveness of this activation was verified qualitatively by attempting WCA measurements immediately after UVO treatment. The water droplets dispersed instantly upon contact with the surface, indicating a highly hydrophilic surface with effective hydroxylation. Due to this immediate spreading, no contact angle could be measured reliably. A representative video of the behavior is included in the Supporting Information (Video V6, referenced in Table S1).

Following activation, the sample was transferred to a vacuum oven (Vacutherm, ThermoScientific) with pressure and temperature control as well as an input for N_2_ gas. A glass container filled with 500 μL of the silane compound (FOTCS, FDDTCS or TMCS) was opened and placed in the chamber next to the sample (Figure 2b). The oven’s chamber was then vacuum purged and flushed with inert nitrogen gas, and then pumped down and heated up to a specific process pressure and temperature. Samples were incubated for 10 min, unless otherwise specified. The low pressure and high temperature promote the evaporation of the silanes, enabling the creation of a self-assembled monolayer of silanes on the sample’s surface. It should be noted that just a small amount of silanes from the vial evaporate, and the vial with the liquid or solid compound can be thus used multiple times. The specific process parameters are listed in Table 1 for the three different silanes used in this work.

**Figure 2:**
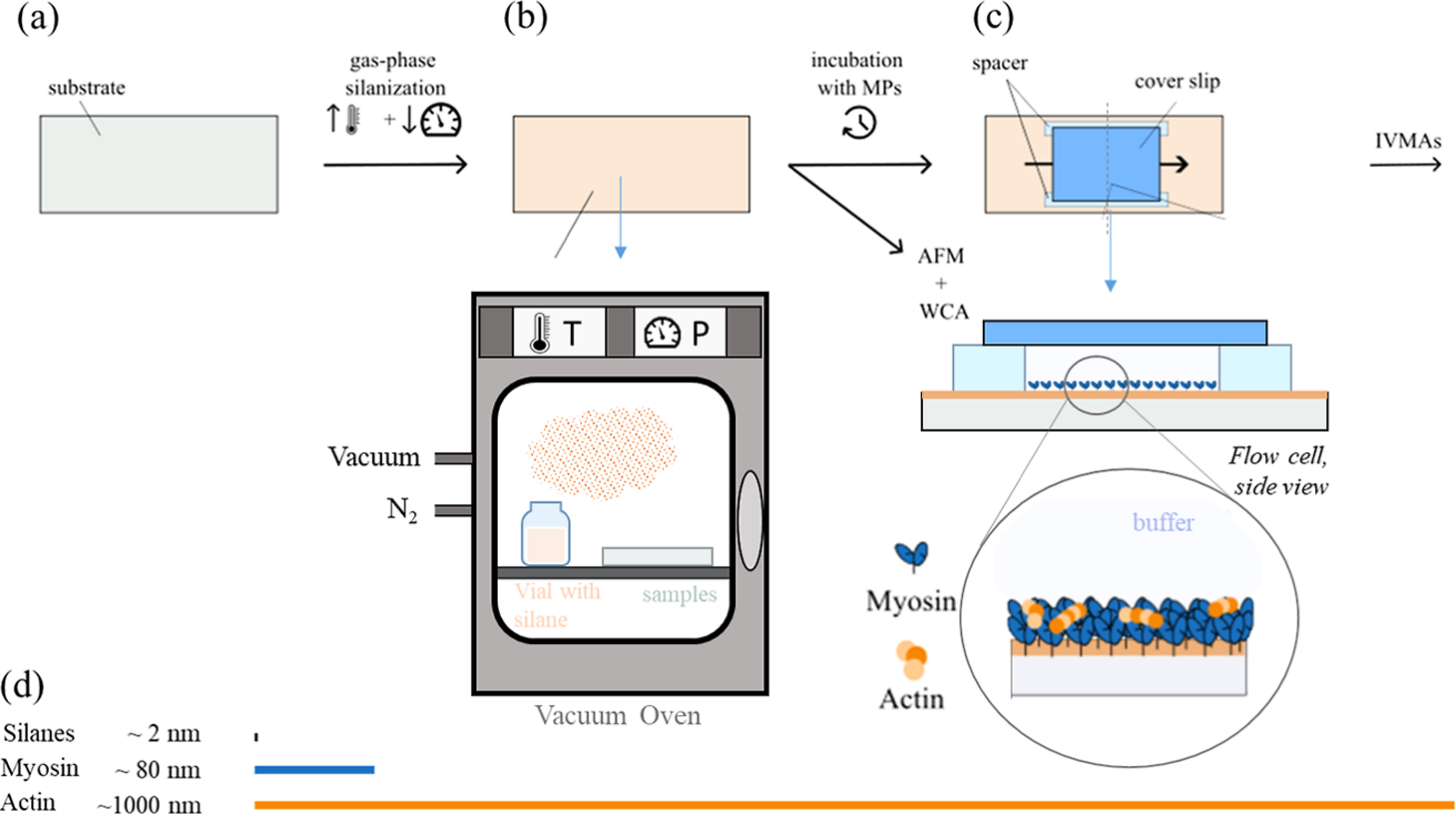
Overview of the general workflow for sample fabrication and data acquisition. (a) Clean, activated glass or polymer substrates are incubated at high temperatures and low pressures for functionalization. (b) Three different silanes (TMCS, FOTCS, FDDTCS) have been used to increase the hydrophobicity of the substrates. The deposition is done inside a commercial, all purpose vacuum oven, with vacuum and N2 connection, and control over temperature and pressure. The samples are placed inside, together with an open vial containing the silane. The substrates are characterized with WCA measurements and AFM to assess the hydrophobicity and roughness, respectively. (c) The substrates are assembled into a flow cell with a glass coverslip at the top, incubated with motor proteins (MPs) and actin filaments and used for IVMAs. A cross-section of the device is also shown (d) Illustrates typical end-to-end lengths of silanes, myosins and actin filaments in relation to each other.^2^

**Table 1:** Summary of the WCA Obtained on Different Substrates (Glass and Polymer (Ormostamp)), Silanized With Three Different Silane Compounds Using Gas Phase Deposition With Different Parameters^a^

substrate	silane	methodt	temperature [°C]	pressure [mbar]	incubation time [min]	Avg WCA [deg]
glass (control)	-	-	-	-	-	48.4 ± 0.9
glass	TMCS	dip-coating	22	-	10	87.5 ± 0.8
glass	TMCS	gas-phase deposition	22	150	15	88.5 ± 0.8
glass	FOTCS	gas-phase deposition	75	400	15	88.1 ± 1.1
glass	FDDTCS	gas-phase deposition	85	200	15	66.1 ± 1.5
polymer (control)	-	-	-	-	-	48.1 ± 1.5
polymer	FOTCS	gas-phase deposition	75	400	15	104.3 ± 1.6

a In all cases, the goal was to obtain a WCA close to 90°, which is known to be optimal for promoting high quality filament motility (high sliding velocities and high fraction of motile filaments). As a reference, the WCA of the bare, non-silanized materials is also listed, as well as the WCA obtained by dip-coating. The WCA is the average obtained from three samples, made independently for each condition, and the error is the standard deviation (see details in Table S2).

### Water Contact Angle Measurements

The static water contact angle (WCA) measurements were performed on a Drop Shape Analyzer DSA25E′ from Krüss, in the ‘sessile drop’ configuration. The drop shape and contact angle for each picture was fitted and measured automatically by the software from the instrument. Each drop on a sample was measured over 60 s at a frame rate of 3 frames per second, i.e. 180 pictures were obtained per sample. These measurements were performed on flat glass and polymer surfaces coated with the different silane compounds and using different coating parameters. For every condition, three individual samples were measured. The average values obtained for each condition are summarized in Table 1.

### Atomic Force Microscopy Measurements

The samples surfaces were characterized by atomic force microscopy (AFM) using a MFP-3D microscope from Oxford instruments. The AFM images were recorded in tapping mode using an Olympus micro cantilever (OMCL-AC160TS-R3) with a force of 5 nN. All images were obtained over an area of 5 × 5 μm^2^ with 256 × 256 pixels. All roughness analyses were performed with the open source software Gwyddion.

### Silanization by Dip-Coating

For dip-coating with TMCS, glass coverslips were dipped in different liquids and dried under N_2_ flow following a series of steps: piranha-solution (5 min, 80 °C), 3× dH_2_O (2 min), methanol (2 min), acetone (2 min), chloroform (2 min), dried with N_2_ (4 min), TMCS 5% v/v in chloroform (10 min), chloroform (4 min), dried with N_2_ (4 min).

### Protein Preparation

Fast skeletal muscle myosin II was isolated from rabbit leg muscles.^15^ Heavy meromyosin subfragment (HMM) was obtained through limited proteolysis using chymotrypsin.^16^ The protein concentration and purity were evaluated using absorbance spectrophotometry and sodium dodecyl sulfate polyacrylamide gel electrophoresis (SDS-PAGE). HMM aliquots were flash-frozen in liquid nitrogen and stored at −80 °C for further use.^17^ We purified Myosin from a euthanized rabbit, using a procedure approved by the Regional Ethical Committee for Animal Experiments in Linköping, Sweden (ref 17088–2020). The rabbit was first anesthetized by intramuscular injection of 0.25 mL Zoletil (active substances: Zolazepam, 6 mg/kg; Tiletamin, 6 mg/kg och Medetomidin, 0.6 mg/kg) and then euthanized by injection of 2 mL of penthobarbital (100 mg/mL) in an ear vein. The Linnaeus University veterinary performed all procedures. No in vivo experiments on live animals were performed. Therefore, the ARRIVE guidelines are not applicable. The work related to use of laboratory animals was performed in accordance with the Animal Welfare act (Swedish law: SFS: 2018:1192) and the guidelines of the Swedish Board of Agriculture overseeing the use of laboratory animals in Sweden. The mentioned laws and guidelines are in accordance with the EU-Directive 2010/63/EU on the use of animals for scientific purposes.

Commercially available G-actin (250 μg, Hypermol, Germany; Cat. No. 8101–04) from rabbit skeletal muscle was used. This G-actin was resuspended in 59.5 μL deionized (DI) water, followed by polymerization by mixing with 6.6 μL Polymix (Hypermol, Germany; Cat. No. 5000–0*). This resulted in an F-actin solution with a concentration of 4.2 mg/mL (100 μM). The actin filaments were labeled with Rhodamine-Phalloidin (Life Technologies Corporation, U.S.A.; Cat. No. R415) as described by Kron et al.^16^ and by Balaz and Månsson^18^ to obtain a labeled F-actin solution with a final concentration of 168 μg/mL (4 μM on monomer basis).^19^

### Fabrication of Microchannels with Topographical and Chemical Contrast

To fabricate microchannels on a polymer with topographical and chemical contrast to its surrounding surfaces, a silanized glass slide was spin coated with Ormostamp (4000 rpm with an acceleration of 100 rpm/s). Ormostamp is a hybrid, inorganic–organic polymer, commercially available from microresist technology GmbH, which contains SiO_2_ groups, and thus allows for silane-based surface chemistry.^20^ Ormostamp is UV curable and solvent-free. Thus, it is often used as active material in nanoimprint lithography, since it can be patterned at the micro and nanoscale.^21–27^ After that, a PDMS stamp with a desired microstructured surface was pressed against the Ormostamp layer and exposed with UV-light (pulsed Xe-lamp, broadband, 2000 mJ/cm^2^, 23 s, EVG nanoimprinter 501) to cure the Ormostamp. After removing the stamp, a residual layer of polymer remained at the bottom of the imprinted microchannels. This residual polymer layer was etched away by inductively coupled plasma reactive ion etching (ICP-RIE) (SI 500, from Sentech) for 6 min with a mixture of oxygen plasma and SF_6_ (RF-power: 50 W, O_2_-flow rate: 60 sccm, SF_6_-flow rate: 10 sccm, reactor pressure: 0.3 Pa), to reveal the silane coated glass surface underneath the polymer. The microstructured polymer coated glass slides were also used as bottom of flow cells for IVMAs.

For motility assays performed on nonstructured polymer surfaces, a glass slide was spin coated with a polymer (Ormostamp) film (4000 rpm with an acceleration of 100 rpm/s), silanized, and used as bottom of the flow cells.

### In Vitro Motility Assays (IVMA)

To assess the compatibility of the actin-myosin system with our silane compounds and the quality of the surfaces coated by gas phase deposition, we performed in vitro motility assays.^10^ For this, we made flow cells using the silanized substrates as bottom, double-sided tape strips as spacers, and a smaller nonfunctionalized glass cover slide as top as sketched in Figure 2c. Buffers for IVMA experiments are prepared with a low-ionic strength solution (LISS) as a base, which contains 1 mM 3-(*N*-morpholino)­propanesulfonic acid (MOPS), 1 mM magnesium chloride (MgCl_2_) and 0.1 mM potassium ethylene glycol-bis­(β-aminoethyl ether) -*N*,*N*,*N*′,*N*′-tetraacetic acid (K_2_EGTA). For wash buffer preparation, LISS was used with addition of 50 mM potassium chloride (KCl) and 1 mM dithiothreitol (DTT) (final concentrations). The assay solution was prepared by mixing LISS buffer with 10 mM DTT, 45 mM KCl, 2.5 mM creatine phosphate (CP), 0.2 mg/mL creatine phosphokinase (CPK), 1 mM magnesium adenosine triphosphate (MgATP), 3 mg/mL glucose, 0.1 mg/mL glucose oxidase (GOX) and 0.02 mg/mL catalase.

For each individual experiment, the flow cells were incubated with HMM and rhodamine labeled actin filaments according to a modified version of a previously described protocol.^8^ The flow cells were incubated in the following steps: 5 min HMM [120 mg/mL], 2 min BSA [1 mg/mL] 2 min rhodamine labeled actin filaments diluted with wash buffer (10 nM for the IVMA performed on the different glass substrates, 20 nM for the IVMA on polymer substrates) followed by assay solution.

All IVMA experiments were performed in a laboratory with controlled temperature and humidity, ensuring stable environmental conditions during sample preparation and data acquisition. Furthermore, each motility assay was conducted within a sealed flow cell, consisting of a functionalized bottom substrate, a glass coverslip on top, and double-sided tape as spacers. This enclosed system isolates the motility buffer from ambient air, minimizing the influence of external humidity on the experiment. Given the short duration of each measurement and the closed nature of the assay environment, environmental humidity is not expected to impact filament motility. All experiments were performed at an ambient temperature of 22 °C. The lab temperature was monitored before each experiment.

### Data Acquisition and Analysis

During the motility assays, the filaments were imaged using an inverted microscope (ECLIPSE Ti–U Nikon) in epifluorescence mode, with a 100× immersion oil objective (NA = 1.45). A metal halide lamp (Insenslight, from Nikon), was used as excitation source, and an sCMOS camera (Sona, from Andor) for imaging. The videos of the motility assays were acquired at 5 frames/s over 40 s. The videos were analyzed with the imageJ plug-in MTrackJ^32^ where individual filaments can be tracked along the time series. The average sliding velocities were then obtained. For every condition, three samples were prepared and measured with tracking of 30 filaments in total (10 filaments per sample).

The recorded videos were additionally used to calculate the average fraction of motile filaments, also looking at three samples per condition. To quantify the fraction of motile filaments (*X*_motile_), we also used ImageJ. In the first frame of each video, all filaments were counted and manually circled. This represents the total number of filaments present at the start of the observation (*N*_total_). After that, we counted all filaments that remained in their original positions during the observed time (40 s), indicating a lack of movement. These filaments are considered nonmotile (*N*_nonmotile_). The fraction of motile filaments *X*_motile_ was then calculated following eq 1.

1 $$X_{motile}=\frac{N_{total}-N_{non‐motile}}{N_{total}}$$

## Results

We have used three different silanes to coat the substrates (Figure 2) and change their hydrophobicity: trimethylchlorosilane (TMCS), perfluoro-octyltrichlorosilane (FOTCS) and perfluoro-dodecyltrichlorosilane (FDDTCS) (Figure 1). TMCS or similar silanes have been used by different groups for IVMA functionalization,^10,12,37,38^ but it presents two main drawbacks. First, it is toxic and highly flammable (Figure S2). Second, the associated standard deposition process based on dip coating in liquid involves using large amounts of organic solvents and harsh reagents like piranha solution (sulfuric acid plus hydrogen peroxide). In addition, the general dip coating procedure is manual, requiring several hours of labor-intensive work. Even more, since many steps are involved, it is challenging to scale up the process to produce high-quality, functional surfaces with reproducibility. Thus, safer, simpler alternative options and processes are desirable.

FOTCS and FDDTCS are alternative silane compounds. They have higher molecular weights and are more stable and less volatile, easing their manipulation in the lab, and reducing the associated risks. Both are less dangerous than TMCS in case of exposure, they are not flammable and are more stable at higher temperatures as reflected by their higher boiling points *T*_b_ (Figure 1). A comparative list of the associated toxicity and handling hazards from the MSDS of the three silane compounds is attached in Supporting Information (Figure S2), built on the most recent toxicology data. In addition, they have long, fluorinated aliphatic chains, which typically result in surfaces with higher hydrophobicity.

Silanization in gas phase allows for a simpler and faster process, where no other chemicals besides the silane itself are required.^10^ The total process time is just around 20 min per run, being much faster than dip coating, which requires several hours. Another advantage of this method is that it is possible to coat multiple samples simultaneously, by placing them inside the oven at the same time. In addition, deposition of silanes in liquid is usually not suitable for coating nanostructures, since the silanes tend to accumulate at corners,^28^ while vapor phase coating has been widely demonstrated as a valid alternative for that purpose.^24^

### Surface Functionalization via Gas-phase Deposition

Glass samples were functionalized using the gas-phase deposition process with the three different silane compounds. The results were first evaluated by measuring the water contact angle of the treated samples for different coating conditions for each silane. We aimed at obtaining substrates with WCA above 80°, since they have been found to support high sliding velocities.^5,6,10^

For the coating of surfaces via gas-phase deposition of silanes, we use a commercial oven where we can purge with N2 to avoid humidity in the chamber (which would have resulted in silane polymerization). The oven also allows for control of pressure and temperature. We insert a small vial with the silanes in liquid form (0.5 mL), which evaporate due to the high temperature and low pressure, and coat the surface of the samples from the vapor phase, typically forming a self-assembled monolayer of silanes, covalently bonded to the surface.^29–32^ There are three parameters that can be used to tune the surface coverage and control the WCA: the process time, the temperature and the pressure. Figure 3a shows the obtained WCA for glass samples coated with FOTCS (black marks) and FDDCTS (red marks) at different temperatures, for a fixed chamber pressure (400 mbar and 200 mbar respectively) and incubation time (10 min). Higher temperatures promote the evaporation of the silanes and thus result in a denser gas, a higher surface coverage, and thus a higher WCA. Figure 3b shows that longer incubation times result in higher WCA, reaching a saturation point at WCA ≈110°. That graph also shows that the low pressure in the chamber promotes the silane evaporation and allows to obtain samples with higher WCA in shorter incubation times, although the effect of the pressure within the range achievable by the oven (between 200 and 600 mbar), is not as pronounced as the effect of obtained by tuning the temperature or the time. We also observed that the method is less reproducible for shorter incubation times - thus, we considered 10 min a trade-off between obtaining samples with consistent WCA without significantly slowing down the process.

**Figure 3:**
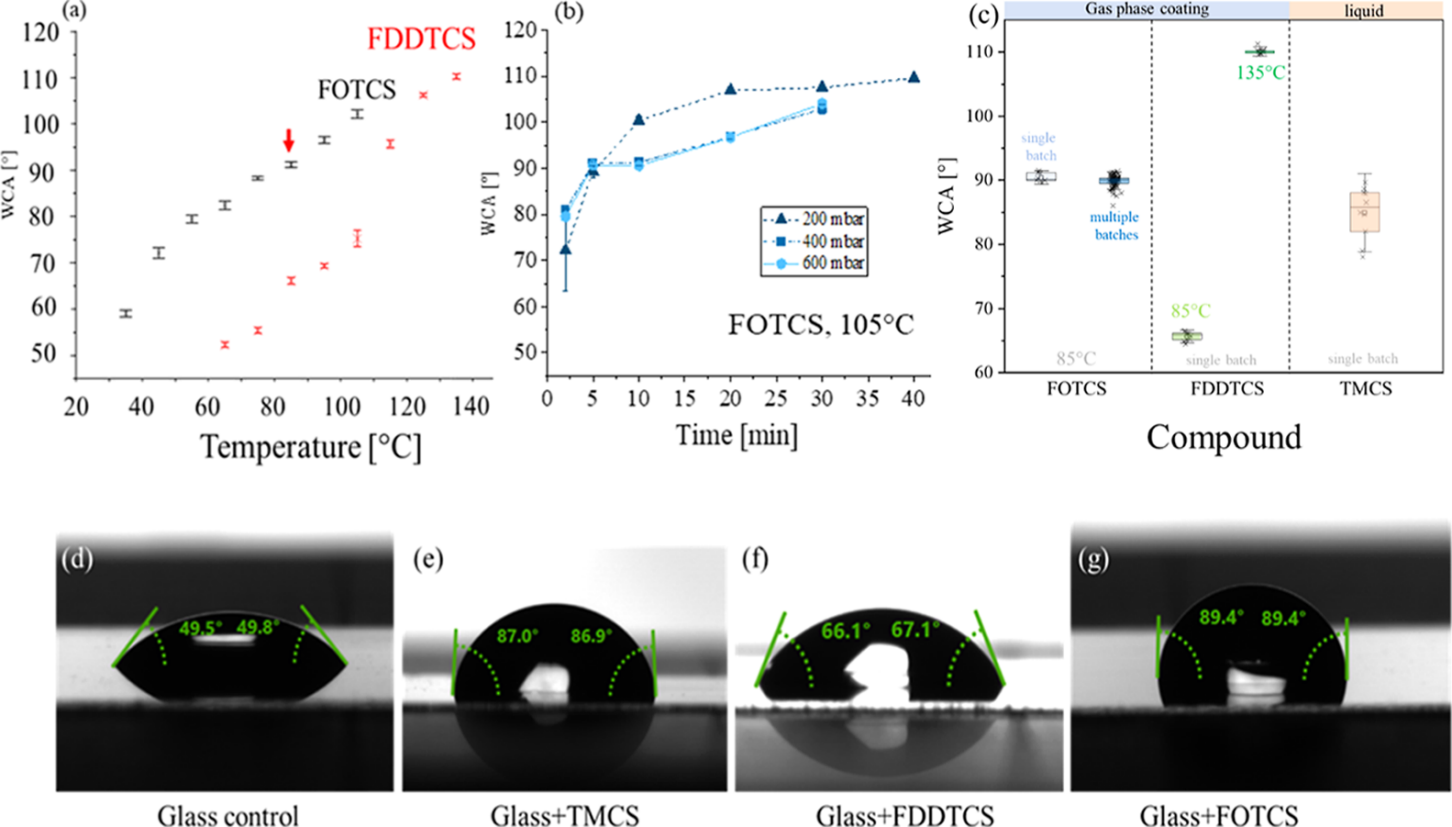
(a) Average water contact angles (WCA) on glass surfaces silanized with FOTCS (400 mbar) and FDDTCS (200 mbar) by vapor phase deposition after 10 min incubation for different process temperatures. (b) Average water contact angles (WCA) for glass surfaces silanization with FOTCS (200, 400, 600 mbar) by vapor phase at 105 °C after different incubation times. (c) WCA measured after functionalization of glass slides with different protocols. Silanization in the liquid phase via dip-coating shows a higher variability in contact angles when compared to silanization with FOTCS or FDDTCS via the gas-phase. For every single batch condition 10 samples were functionalized and measured. (d) Water contact angle (WCA) measurements on clean and silane-functionalized glass surfaces with TMCS (e), FDDTCS (f) and FOTCS (g). Measurements were made using 20 μL water droplets over a period of 60 s at 5 fps.

We have observed that FDDTCS, which has a longer chain and is thus more stable at higher temperatures, is more difficult to evaporate, so higher temperatures and lower pressures are needed to achieve hydrophobic surfaces. At similar process parameters, the obtained WCA after FDDTCS silanization are lower than those obtained for FOTCS. To obtain the targeted WCA above 85°, temperatures above 110 °C are needed, as shown in Figure 3a. As many applications in nanotechnology use polymer substrates and polymer micro/nano structures, we decided to keep the process temperature below 90 °C to ensure the overall structural integrity of the devices and compatibility with all materials used.

Thus, for the IVMA, and to compare our results with those previously reported in the literature, we used glass slides coated with TMCS and FOTCS with a WCA close to 90° (88.5° ± 0.8° for those coated with TMCS, and 88.1° ± 1.1° with FOTCS), and glass samples coated with FDDTCS with a lower WCA (67.6° ± 1.5°). As a reference, the WCA on nonsilanized control glass samples is 48.4° ± 0.9°. A summary is shown in Figure 3c–g and Table 2.

**Table 2:** Summary of the Different Materials Used as Underlying Substrates, Silanes for Coating, Water Contact Angles (WCA), Root Mean Square Roughness (RMS), Observed Average Velocity and Ratio of Motile Filaments Obtained during IVMAs^a^

material	silane	Avg WCA [deg]	RMS roughness [nm]	Avg velocity at ambient temperature [μm/s]	Avg ratio of motile filaments [deg]
glass	uncoated (control)	48.0 ± 0.9	0.97	-	-
glass	TMCS (dip-coated), literature^5,9^	86	-	1.9	92
glass	TMCS (dip-coated), this work	85.0 ± 3.9	-	3.2 ± 0.5	90.9 ± 0.9
glass	TMCS	88.5 ± 0.8	-	3.3 ± 0.4	90.4 ± 0.7
glass	FOTCS	88.1 ± 1.1	1.39	3.9 ± 1.2	86.6 ± 1.8
glass	FDDTCS	66.1 ± 0.9	1.83	2.6 ± 0.9	67.6 ± 2.3
polymer	uncoated (control)	48.2 ± 0.9	1.30	-	-
polymer	FOTCS	104.3 ± 1.6	2.80	3.1 ± 0.2	69.8 ± 1.5

a 10 samples were coated within one batch. Errors represent the standard deviation from the mean. IVMAs were performed at *T* = 22 °C.

To systematically assess the reproducibility of the method, we performed coatings using batches of 10 samples under various conditions (Figure 3c). For each condition, we analyzed the dispersion in water contact angle (WCA) within individual batches. Additionally, we performed WCA measurements on three samples that were fabricated in independent processes, which are listed in Table S1. Since we obtained the target WCA of 90° using FOTCS (at process temperatures applicable to our material system), we extended the reproducibility analysis by compiling WCA measurements from up to 90 samples, each coated in separate batches on different days over the course of a year (Figure 3c, dark blue markers). This data demonstrates the long-term reproducibility of the method.

The surface of the silanized samples was investigated using an AFM (Figure 4, Table 2). Untreated glass surfaces (Figure 4a) show an rms roughness of 0.97 nm. The AFM images showed an increased roughness on the FOTCS (c) (1.39 nm) and the functionalization with FDDTCS yielded the highest roughness on glass samples, with an rms of 1.83 nm (k). Rougher hydrophobic surfaces increase the contact surface between liquid and solid, which leads to higher surface energy and thus to an increased WCA^19^ (Figure 3c–f). Fluorinated silanes such as perfluorinated compounds can have sterically bulky structures, which sometimes can lead to difficulties in forming a monolayer, producing clusters upon adsorption (see discussion below). This is clearer for the FDDTCS samples, which show larger clusters due to higher molecular weight.

**Figure 4:**
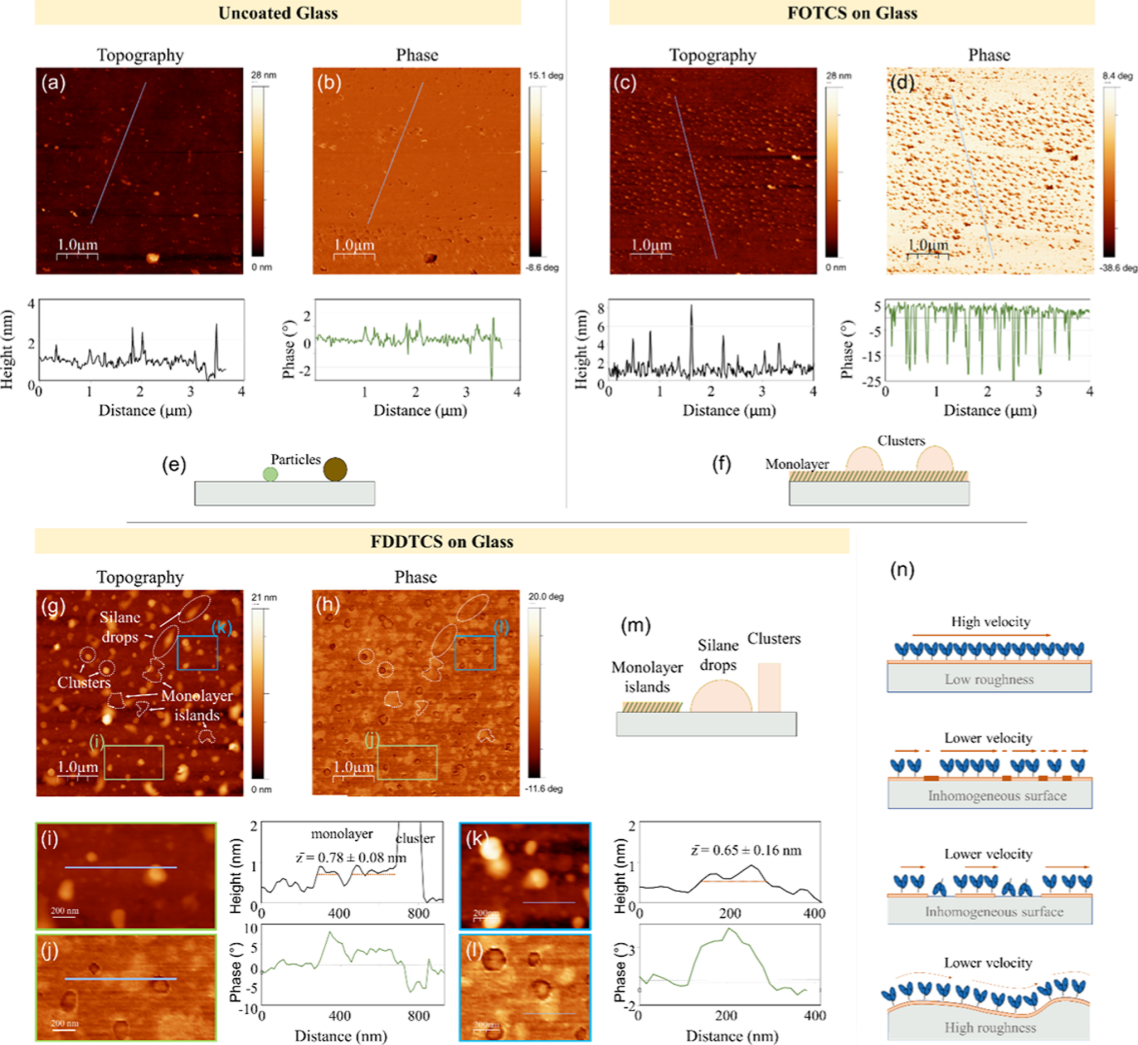
Atomic force microscopy (AFM) images of glass surfaces functionalized with different silanes. Topography (a) and phase image (b) of a glass surface without silane coating as control (RMS = 0.973 nm) with corresponding profiles, plotted from the lines marked in the images. Topography (c) and phase image (d) of a Glass surface functionalized with perfluoro-octadecyltrichlorosilane (FOTCS, RMS = 1.39 nm) with their profiles, respectively. (e,f) show sketches or our interpretation of the images for uncoated and FOTCS coated samples, showing that on glass, there are small contamination particles on the surface, on the FOTCS coated samples, we obtain a monolayer with small droplet shaped clusters on top. Topography (g) and phase (h) of a glass surface functionalized with perfluoro-dodecyltrichlorosilane (FDDTCS, RMS = 1.83 nm). Details of different areas are shown in (i,j) and (k,l), obtained from the regions marked in (g,h). Together with corresponding profiles. On the FDDTCS coated samples, there are monolayer islands, silane wetting drops, and clusters, all three structures with distinct topography and phase contrast signals. (m) Sketch of our intepretation of the images for FDDTCS. (n) Schematic illustration of the possible effect the surface might have on the sliding velocity: an ideal system with very low roughness and homogeneous surface chemistry will result in dense motor protein coverage, with homogeneous distance between them, with filaments propelled at high velocities. Inhomogeneous surfaces will result in lower coverage with motors, with different distances between them, thus reducing the propelling efficiency and reducing the velocity. If this inhomogeneous surface is composed of hydrophilic and hydrophobic regions, this will result in motors adsorbed with the actin-binding site facing the surface or so-called “dead-heads”, resulting in surfaces with lower fraction of motile filaments, which get stuck at these spots. Rough surfaces result in motors adsorbed with different spacing and with different orientations, thus having a negative impact on the motility.

The topography images (Figure 4c,g) together with the phase images (b,d,h) can help us understand the monolayer formation and surface coverage on the different samples. In the FDDTCS images, we observe prominent nanoscale pillars (10–20 nm height) along with flat terraces separated by subtle steps of approximately 0.6–2 nm. These features, consistent with the expected thickness of self-assembled fluorosilane monolayers, suggest the formation of discrete silane islands (Figure 4m). The corresponding phase images displayed clear contrast between these regions: areas with no measurable step height exhibited a phase signal close to 0°, while regions raised by 1–2 nm showed increased phase values (∼5°). Two representative examples of topography and phase images and their profiles can be seen in Figure 4i,j,k,l, to illustrate this statement. The shift in phase is attributed to changes in tip–sample interactions due to the presence of the fluorinated organic layer, indicating reduced adhesion relative to the bare glass substrate. In contrast, the FOTCS samples have a more uniform topography with a continuous baseline and widespread nanoscale clusters (5–10 nm in height). The phase image of this sample revealed a homogeneous signal (∼5°) across the base surface, suggesting a uniform fluorosilane coverage (f). Interestingly, the phase decreased sharply to approximately −20° over the clusters, implying a change in material properties, such as increased stiffness, energy dissipation, or molecular packing density within these domains. As a reference, uncoated glass samples showed a flat topography and negligible phase contrast (Figure 4a,b).

### In Vitro Motility Assays on Glass Samples Functionalized via Gas-phase Deposition

After achieving adequate hydrophobicity on the sample surfaces, IVMAs were performed to assess the compatibility of the proposed material systems with motile function of the acto-myosin system. The glass surfaces functionalized with the different silanes were coated with the myosin motors and used as part of a flow cell (Figure 2c) to study the motility of actin filaments (fluorescently labeled) propelled on top (see Videos V1, V2, V3, V4 and V5 in Supporting Information). The surfaces were imaged by epifluorescence microscopy, and the recorded videos were used to track filament movements and determine average sliding velocity. The measurements were repeated on three individual samples for each material system, and the obtained velocities were averaged. Figure 5a shows a frame of a video, with several actin filaments of different lengths being propelled in different directions. In the image, we have marked example motion paths for different filaments obtained using the MTrackJ plugin^32^ in ImageJ. Figure 5b shows a box plot of average sliding velocities of filaments on glass coated with the three different silanes in gas phase. For each condition, we performed IVMAs on three different samples and measured the sliding velocity of 10 filaments on each. To check for reproducibility of these results, we made a new batch of samples coated with FOTCS, motors and filaments, and performed the experiments again, and measured 10 more filaments on three more samples (see the details in Figure S4 in the Supporting Information). Figure 5c shows the average ratio of motile to nonmotile filaments measured from the same videos. Filaments on glass coated with FOTCS showed the highest average sliding velocity, which was 3.9 μm/s ± 1.2 μm/s. Additionally, in these samples we observed a high percentage of motile filaments (87.0% ± 1.8%). For reference, we have observed that bare glass does not support motility. On untreated glass, the filaments just adsorb locally to the substrate (i.e., some parts of the filaments adsorb, while the rest floats in the liquid). These observations are consistent with findings in the literature.^5,9^ The measured 0 μm/s velocity and absence of motile filaments confirm the necessity of silane coating. ANOVA (Analysis of Variance) tests showed that even if the variance within sample populations is wide, there is a statistically significant variation between groups (*F*(2,117) = 19, *p* = 7 × 10^–8^) (see details in Table S2). When the IVMA were repeated using three different samples prepared under similar conditions and measured within a few days, no statistically significant variability was observed (*F*(2,27) = 0.001, *p* = 0.999). We also observed that repeating the measurements several months later using FOTCS to coat Ormostamp and performing the IVMA with different motors and filament batches resulted, in statistically significant differences (*F*(1,58) = 4.6, *p* = 0.036). However, the effect size is small (Cohen’s *f* = 0.28), suggesting that although the differences are statistically significant, the practical impact is limited (see data comparison side by side in Figure S4). For TMCS, an almost monotonic correlation between contact angle and velocity has been reported.^5,10^ For FOTCS we observed the same trend, as shown in Figure 5d: samples with higher WCA (>85°) yielded higher average velocities, with the highest sliding velocity obtained for samples with a WCA ≈110°, which is the highest WCA that we could obtain. Notably, here, the dispersion in the data is also smaller, since we believe that a more packed silane layer allows for a more homogeneous and dense coating of motors with the optimal orientation (Figure 4n).

**Figure 5:**
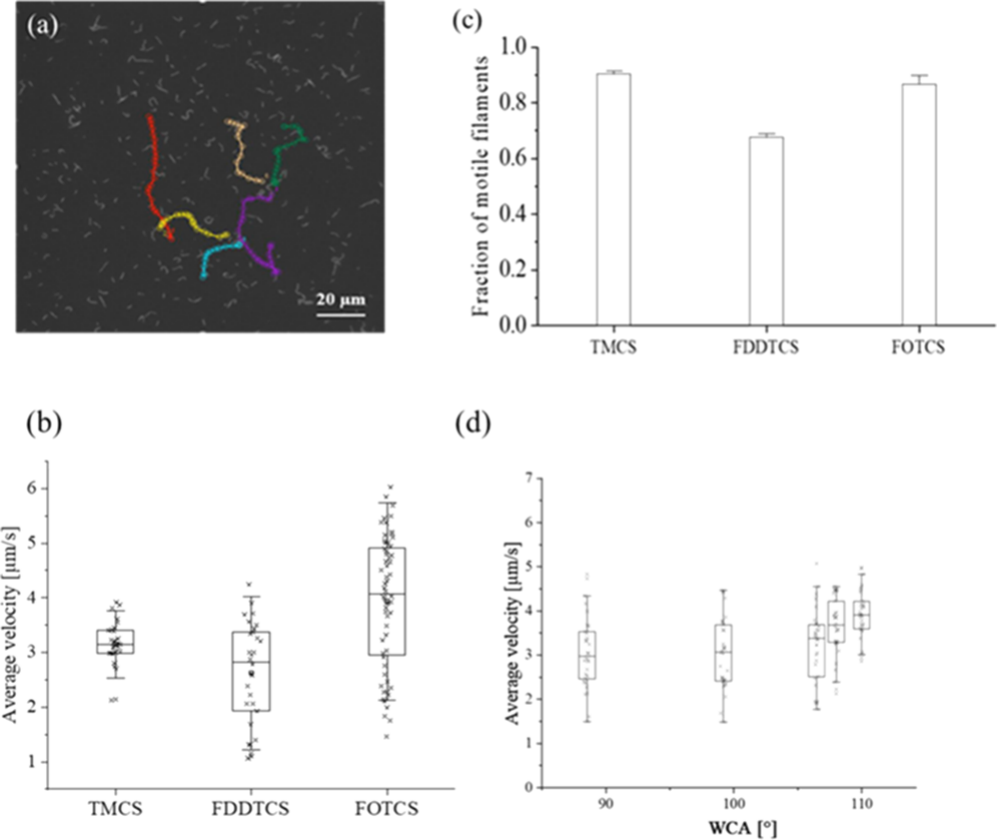
(a) Motility analysis of filaments via evaluation of sliding tracks of individual actin filaments performed in ImageJ (using the plugin MtrackJ). (b) Box-plot of average sliding velocities of 30 (TMCS, FDDTCS) or 60 (FOTCS) actin filaments,where the box gives the middle two quartiles of the data and the central line represents the median. Note: FOTCS and FDDTCS follow a similar trend to TMCS (higher WCA lead to higher filament velocities). (c) Fraction of motile filaments measured on glass surfaces functionalized with three different silane compounds using the gas phase deposition method. Data and error bars in (c) represent the standard deviation from average of three individual samples. IVMA performed at 22 °C. (d) Box-plots of sliding velocities of actin filaments on samples coated with FOTCS at different WCA (30 filaments per condition). Details on the reproducibility of the measurements across different samples, motor and filament batches are shown in the Supporting Information (Figure S4).

Overall, these results are comparable to those obtained in other publications using glass samples dip coated with the conventionally used silane, TMCS. In those works, sliding velocities in the range from 2 to 8 μm/s have been measured at various temperatures (19–30 °C), where higher temperatures lead to higher velocities.^6,9,10,12^ For the samples measured in our lab at 22 °C, the average sliding velocity on TMCS-functionalized surfaces was 3.3 μm/s ± 0.4 μm/s, having a high fraction of motile filaments (90.4% ± 0.7%).

Samples coated with FDDTCS showed a lower sliding velocity of 2.6 μm/s ± 0.9 μm/s compared to the samples coated with the other two compounds, and the fraction of motile filaments also decreased down to 67.6% ± 2.3%. This might be due to the fact that these samples are less hydrophobic (as shown by the lower contact angle), and/or the higher roughness of the surface and the lower surface coverage after functionalization with FDDTCS, (Figure 4k). As observed, the surface is not densely coated with the silanes, but there are islands of silanes, with some nanoscopic areas which are coated with lower densities of silane molecules, or even locally with none. In these regions, the motor proteins were probably exposed to a naked, hydrophilic glass surface, and they most likely bind with their actin binding region (head domain) to the surface in contrast to binding via the desirable tail domain. This would result in lower motility of the filaments due to nonfunctional “dead” heads or fewer active motors per unit area. In the same line, the samples with the highest silane coverage, and thus, the highest WCA, also showed the highest average sliding velocities.

### Motility on a Flat, Functionalized Polymer Surface

Motility on surfaces that are easy to structure, transparent to visible light and with chemical/topography contrast would be quite useful in applications in nanotechnology. The work of Reuther et al.^7^ has already shown that fabricating three-dimensional, polymeric, motility promoting microtracks is possible for the kinesin motor system. This has not been achieved for the HMM motor system yet. Thus, we also performed actin-myosin motility tests on polymer surfaces, functionalized with FOTCS by gas phase deposition. The polymer used in this study (Ormostamp) is a hybrid polymer composed of inorganic Si- and organic C-groups. The silicon content makes this polymer compatible with silane surface chemistry to adjust the surface’s properties (e.g., hydrophobicity, surface charge, functional groups or roughness) to meet application-specific requirements. Additionally, it is UV-curable and solvent free, which makes it especially suitable for the fabrication of micro- and nanostructures, as shown in previous works.^20–25^ FOTCS has been used to adjust the polymer surface hydrophobicity as it yielded the highest sliding velocities while retaining a high fraction of motile filaments on glass surfaces.

First, the successful functionalization of the polymer surface was verified via WCA measurements and the roughness of the surface was checked by AFM (Figure 6). The nonfunctionalized polymer sample showed an average WCA of 48.2° ± 0.9°, while the samples functionalized with FOTCS showed a WCA of 104.3° ± 0.9°. The roughness of the polymer surface also increased after silanization from an rms of 1.3 nm up to 2.8 nm. During IVMAs (Figure 7c), an average sliding velocity of 3.1 μm/s ± 0.2 μm/s was observed, which is comparable to the values measured on glass samples coated with the same silane (Figure 5). We repeated the measurements several months with different motor and filament batches, and observed very good reproducibility (see data comparison in Figure S2). ANOVA showed no significant variability in the measured filament sliding velocities in the two sets of experiments (*F*(1,58) = 2.3, *p* = 0.13).

**Figure 6:**
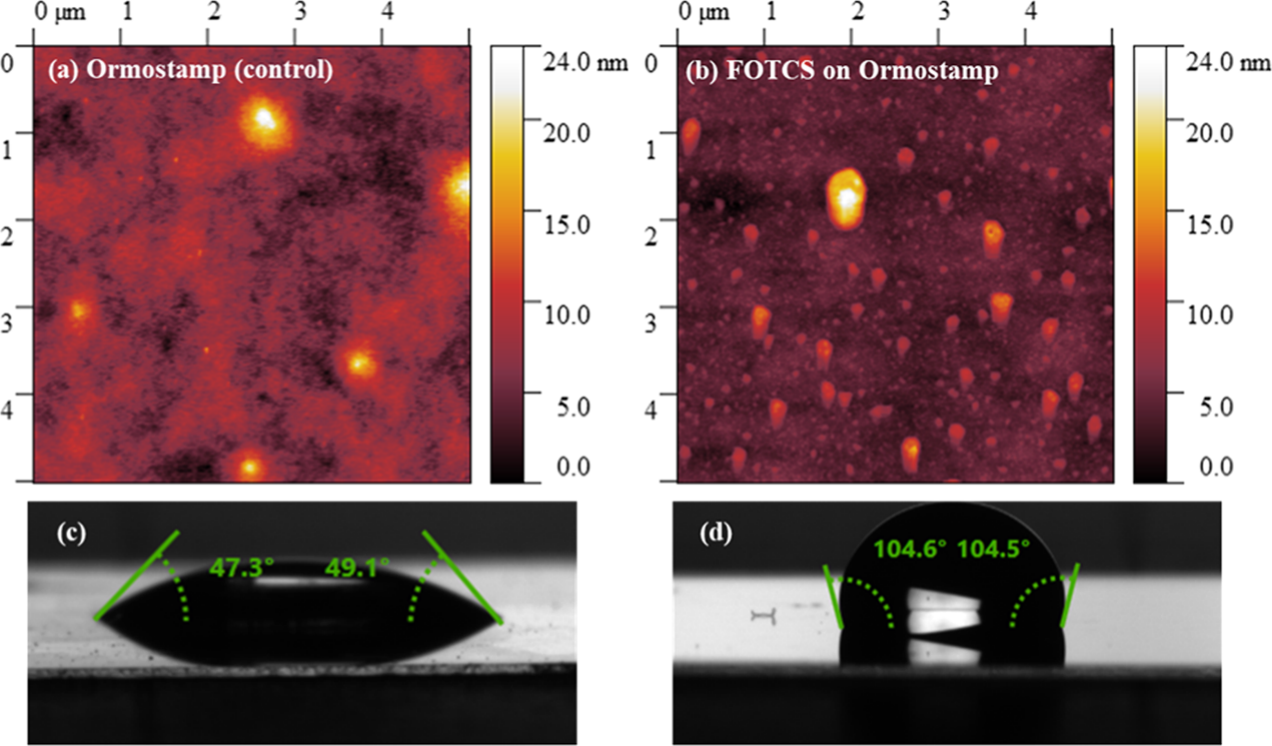
(a) Atomic force microscopy (AFM) images of Ormostamp (polymer) surfaces without coating (RMS = 1.3 nm) and (b) functionalized with FOTCS (RMS = 2.8 nm). We attribute observed vertical features to nanoclusters of adsorbed silane molecules. (c) WCA measurements on an Ormostamp surface without coating and (d) after functionalization with FOTCS.

**Figure 7:**
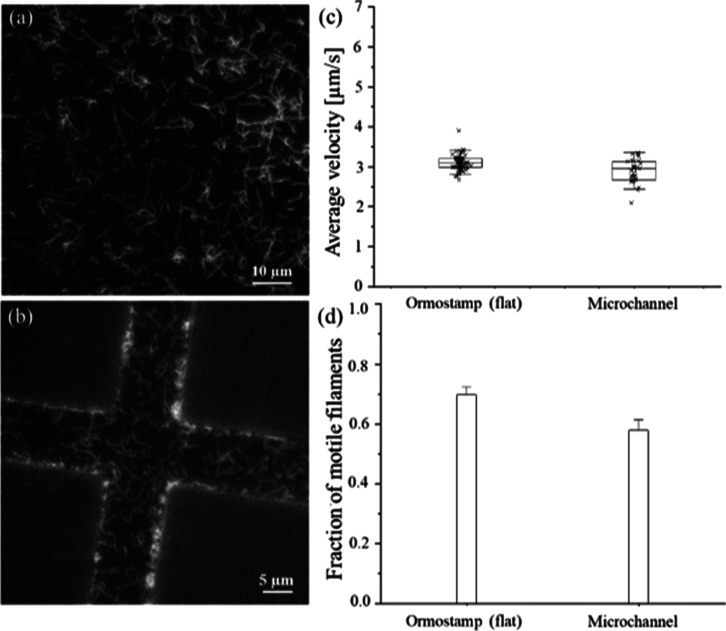
(a) Epifluorescence image of rhodamine-phalloidin labeled actin filaments propelled by myosin motors adsorbed on a flat, transparent hybrid polymer surface (Ormostamp) functionalized with FOTCS in a vapor phase. (b) Motility of actin filaments on the floor of glass functionalized microchannels imprinted into Ormostamp. The Ormostamp walls are areas outside the microchannels that have not been functionalized. Thus, there is no filament motion on them. (c) Box-plot of average sliding velocities of *n* = 60 individual actin filaments on flat Ormostamp polymer surfaces and *n* = 30 on imprinted microchannels where the box gives the middle two quartiles of the data, the central line represents the median. (d) Fraction of motile filaments measured on flat Ormostamp polymer surfaces and inside imprinted microchannels. Error bars in (d) represent the standard deviation from the mean of three individual samples. IVMA performed at 22 °C. Details on the reproducibility of the measurements across different sample, motor and filament batches are shown in the Supporting Information.

The fraction of motile filaments, decreased to 69.6% ± 1.5% in these samples. The reduction in sliding velocity and motile fraction on the polymer compared to glass is likely due to the increased surface roughness after silanization, as seen in the AFM measurements (see Figure 6). Higher roughness may disrupt optimal myosin adsorption or introduce nanoscale barriers to filament movement (see sketch in Figure 4n). Nevertheless, a substantial fraction of the motors is active on such material systems. These values indicate that it will be possible to use HMM motor system on three-dimensional, transparent, polymeric structures made from Ormostamp in the future.

### Filaments in Microchannels in a Hybrid Material System

As a proof-of-concept of confined filament motion, we used hydrophilic polymer microchannels with a hydrophobic glass bottom to perform the motility assays. The microstructures were imprinted on glass slides, previously spin coated with Ormostamp. The glass slides had been functionalized with FOTCS before the coating. In this way, we create a topographical relief with chemical contrast between the hydrophobic channel floors and their surrounding polymer walls. (Figure 7c). To test the applicability of this approach for its use in micro and nanodevices, we performed IVMAs with the aim to observe selective binding and motility confined to the channel floors, but not on the surrounding polymer surfaces (Figure 7c). While the fraction of motile filaments decreased to 57.9% ± 4.4% compared to glass samples (87.0% ± 1.8%), we could observe a strong contrast between channel floors and surrounding polymer surfaces, where filaments predominantly bound to the desired regions. This is reflected in the number of filaments per unit area, that were evaluated in Figure S2. On the polymer area outside the microchannel, a total of 126 filaments in 12600 μm^2^ were counted, which corresponds to a density of 0.009 filaments/μm^2^ (Figure S2c,d). On the functionalized channel floors 170 filaments in an area of 2140 μm^2^ were counted, which corresponding to a density of 0.08 filaments/μm^2^, i.e. an order of magnitude higher than on the areas outside the channels.(Figure S1a,b). The average filament sliding velocity on channel floors was 2.9 μm/s ± 0.3 μm/s, which is comparable to the values obtained for similar, flat glass surfaces (Figure 7b). The ability to control motility-promoting and motility-inhibiting regions is highly important for the integration of the actomyosin system in nanodevices. Combining the topographical structuring together with the potential to use these motor systems on three-dimensional, polymeric structures,^21–23^ a variety of applications in sensing, Lab-on-a-chip devices and biocomputation technologies could be explored in the near future.^5^

## Discussion

The results presented here demonstrate that gas-phase silanization is a powerful, simple, and versatile method for coating a variety of surfaces with different silanes, with very high reproducibility over several batches compared to conventional liquid phase protocols as shown in Figure 3c. In addition, the use of an all-purpose commercial vacuum oven significantly improves the reproducibility of the results compared to previous studies that relied on custom-made setups for the deposition in gas phase.^10,12^ Furthermore, using a vial with liquid silane instead of pipetting or injecting it enables precise control over the silane concentration in the atmosphere, as it depends solely on process time, pressure, and temperature. This eliminates variability from the injected amount of material Additionally, performing the method at high temperature reduces the silane consumption per run. This advantage is particularly beneficial for mass production, as up to 200 samples can be coated simultaneously without increasing material usage or extending the process time. By tuning the pressure, temperature and process time, it is possible to obtain samples with a variety of WCA, reaching WCA as high as 110°. The main factors influencing the result are the incubation time and the temperature, while the pressure plays a minor role, since, for a given *t* and *T*, changing the pressure from 200 mbar to 600 mbar (minimum and maximum achievable by the oven), we have observed a variation of the WCA of 10° or lower.

The results presented here highlight the nuanced relationship between surface chemistry, hydrophobicity, and protein function in actomyosin-based motility systems. While TMCS remains a standard silane for in vitro motility assays, our findings demonstrate that FOTCS can match or even exceed its performance in terms of sliding velocity, while offering safer handling and vapor-phase compatibility with both glass and polymer substrates.

We observed a superior performance of FOTCS over FDDTCS (while both being fluorinated and long-chained) for the deposition conditions used here. One of the main limiting factors was that we decided to use only deposition temperatures below 100 °C, to keep the process compatible with typical polymers used in micro and nanofabrication, like PMMA or polycarbonate, which have glass transition temperatures around 100 °C. AFM characterization indicates that at these process parameters, FDDTCS creates a more heterogeneous morphology, resulting in incomplete monolayer formation and with clusters and silane drops on the surface. These nanoscale features lead to suboptimal myosin orientation or local adsorption of the actin-binding domain (Figure 4n), reducing motility, as observed. The broader spread in velocities for FOTCS and FDDTCS (Figure 5b) likely reflects local heterogeneities in silane packing density and roughness rather than intrinsic motor instability (Figure 4n) The broader spread in velocities for FOTCS and FDDTCS (Figure 5b) likely reflects local heterogeneities in silane packing density and roughness rather than intrinsic motor instability (Figure 4n). The maximum contact angle achievable by TMCS coating of glass substrates (∼90–100°) is smaller than that by FOTCS (∼100–120°). When we target 90 deg WCA for FOTCS coating for the sake of comparison to previous works, probably the surface coverage with silane is not uniformly distributed, with patches of silanes and small areas of bare glass. Because of that, the density of functional motors is lower, and the motility is also lower and less uniform on the surface. In these glass samples covered by FOTCS, full coverage is reflected by a WCA close to 110°. And, because of that, the higher the contact angle, the better the motility, and lower dispersion in the data, since the surface is more homogeneous. The reproducibility analysis (Figure S4) shows this variability remains within expected experimental variance for IVMAs. Yet, our findings align with prior observations that hydrophobicity strongly influences motor performance.^33,34^

On polymer substrates, we observed reduced fraction of motile filaments and lower sliding velocity, which correlates with increased surface roughness. This suggests that roughness thresholds may exist beyond which motor orientation or spacing is compromised. However, the fact that motility is retained at functional levels even on rougher polymer surfaces shows the robustness of the actomyosin system and its potential for polymer-compatible nanofabrication. Additionally, even with lower motile fractions in these confined regions, the system can still perform its intended transport function. A contrast ratio of 10:1 between motility-promoting and nonmotility regions ensures sufficient selectivity for micro- and nanofluidic device operation. Comparable selectivity has been reported for kinesin systems.^7^

Our findings collectively show that temperature vapor-phase silanization is not just a safer alternative, but a useful and tunable approach for functionalizing a variety of substrates in hybrid polymer applications. While gas-phase silanization itself is an established technique,^29,30^ its temperature-assisted, high-throughput implementation using a low-cost commercial oven for actomyosin-compatible surfaces (and its demonstrated compatibility with UV-curable polymers) has not been reported before. Moreover, since the protocol is based on a low cost, all-purpose commercial oven, this could help other research groups to adopt it and standardize their surface preparation procedures.

## Conclusions

We have demonstrated a temperature-assisted gas-phase silanization protocol that allows functionalization of both glass and polymer substrates using TMCS, FOTCS, and FDDTCS. This method yields surfaces compatible with actin-myosin motility assays, with FOTCS-coated surfaces supporting the highest filament velocity and motile fraction. The approach enables effective tuning of surface hydrophobicity while maintaining compatibility with heat-sensitive substrates, including structured polymers. We also show that microchannel patterning can spatially confine filament motion, highlighting the method’s potential for guiding molecular transport in nanodevices. These results provide a scalable and safer alternative to conventional silanization protocols. In addition, proving the compatibility of the method with UV-structurable polymers supporting filament’s motility paves the way for integrating these surfaces into more complex fluidic systems.^6,20,21^ Further exploration of these functionalized surfaces in diverse nanotechnological contexts promises to further increase the applicability of myosin-derived motor systems in chip-based nanodevices.

## Supplementary Material















## Notes

Optimization of gas-phase silanization parameters (temperature, pressure, incubation time). Water contact angle (WCA) measurements on glass and Ormostamp substrates. Fabrication and characterization of microchannels with chemical and topographical contrast. Fluorescence microscopy of actin filament binding and motility in microchannels. Toxicity and handling hazards of TMCS, FOTCS and FDDTCS, including MSDS excerpts. Reproducibility of in vitro motility assays on glass and Ormostamp. Statistical significance analysis of filament velocity data. Lists of submitted video files and MSDS documents. Raw WCA and filament velocity data used for Figures 3, 5, and 7 (PDF)
FOTCS on glass (AVI)
FOTCS on glass (AVI)
FDDTCS on glass (AVI)
FOTCS on ormostamp (AVI)
FOTCS on glass with traced filaments (AVI)
WCA measurement @2.5 minutes of UV-Ozone treatment (AVI)
