# Supplementary material for: Temperature-Assisted Gas-phase Silanization Using Different Silanes for Actomyosin-Based Nanodevices
Source: ACS Omega. 2026 Jan 30;11(6):9630–41. doi: 10.1021/acsomega.5c09878 (PMC12917835; doi:10.1021/acsomega.5c09878)
Supplement: Supplementary file 1 [file ao5c09878_si_001.pdf]

Supporting Information

Temperature-Assisted Gas-Phase Silanization Using Different Silanes for Actomyosin-Based Nanodevices

Tim Erichlandwehr[*1,2], *Jeremy P. Teuber*[1,3], *Rukan H. Nasri*[1,4], *Cagla Selalmaz*[1],*Marko Usaj*[5], *Alf Månsson*[5], *Irene Fernandez-Cuesta*[1,4]

[1] Institut für Nanostruktur- und Festkörperphysik, Universität Hamburg, Luruper Chausseee 149, 22761 Hamburg, Germany

[2] Deutsches Elektronen-Synchrotron (DESY), Notkestraße 85, 22607 Hamburg, Germany

[3] Max Planck Institute for the Structure and Dynamics of Matter, Luruper Chaussee 149, 22761 Hamburg, Germany

[4] Hamburg Centre for Ultrafast Imaging, Notkestraße 85, 22607 Hamburg, Germany

[5] Department of Chemistry and Biomedical Sciences, Linnaeus University, Norra vagen 49, 39182 Kalmar, Sweden

e-mail: tim.erichlandwehr@desy.de, ifernand@physnet.uni-hamburg.de

Optimization of silanization process parameters:

To optimize the silanization process and achieve specific water contact angles (WCAs), around 90° on glass surfaces, we systematically varied the reaction temperature and incubation time while maintaining other key parameters constant. For each deposition condition, three independent samples were prepared and characterized to assess the reproducibility. **Fig. 3 (a)** shows the dependence of the WCA on the deposition temperature, measured on glass for two different silane compounds. There is a clear dependence of the WCA on the silanization temperature: at higher temperatures, more silane is evaporated and a denser atmosphere formed in the oven, which in turn leads to a surface with higher coverage of silanes, and thus a higher WCAs and increased hydrophobicity. By tuning the silanization temperature within this range, it was possible to precisely control the WCA. For further experiments a process temperature of 75°C was used for coating FOTCS, to reach similar WCA values as used in the literature.[5,6]
The deposition of FDDTCS was more challenging, since it is more stable and less volatile than the other two compounds. **Fig. 3** illustrates the average WCA values obtained for silanization with FDDTCS at 200 mbar and FOTCS at 400 mbar at different temperatures. To prevent the melting of polymer chips, samples for motility experiments were silanized at 85°C.

Additionally, the effect of varying incubation times on the measured WCA during silanization was investigated, as shown in **Fig. 3 (b)**. No significant increase in WCA was observed beyond 10 minutes of incubation, therefore no longer incubation times were applied for making the samples used in subsequent motility experiments.

Contact angle measurements:

Tab. S1 WCA measured on several different samples functionalized with the proposed gas-phase deposition protocol for the samples used for the motility assays. For each material system (i.e., substrate material with a specific silane coating), three different samples (repetition #1, #2 and #3) were prepared and measured. Each of these sample repetitions was prepared in an independent process. For each sample, the WCA was measured 180 times – the table shows the average and standard deviation for each set of 180 measurements for each one of the sample repetitions.

Sample Repetition	TMCS on glass	FOTCS on glass	FDDTCS on glass	Glass (control)	Ormostamp (control)	FOTCS on Ormostamp
#1	86.8°±0.3°	89.5°±0.2°	66.8°±1.1°	49.9°±0.3°	49.8°±1.2°	104.3°±0.9°
#2	88.3°±0.8°	89.6°±0.4°	65.1°±0.8°	49.3°±0.6°	47.3°±0.8°	103.8°±1.2°
#3	88.4°±0.6°	85.4°±1.3°	66.5°±0.9°	48.9°±0.7°	48.4°±0.6°	104.9°±0.6°
Avg	87.3°±0.6°	88.1°±0.6°	66.1°±0.9°	49.3°±0.5°	48.5±0.9°	104.3°±0.9°

Fabrication of microchannels with chemical and topographical contrast:

We patterned a glass substrate, coated with FOTCS, with microchannels, where the microchannel walls were made of Ormostamp. We observed a 10 times larger coverage of filaments inside the glass microchannel floors than on the surrounding polymer surfaces, as expected (see **Fig. S1**). In addition, the filaments on the channel floors were motile (see main text for details), while those on the polymer walls were not mobile, probably due to the motors adsorbing with the head on the surface.

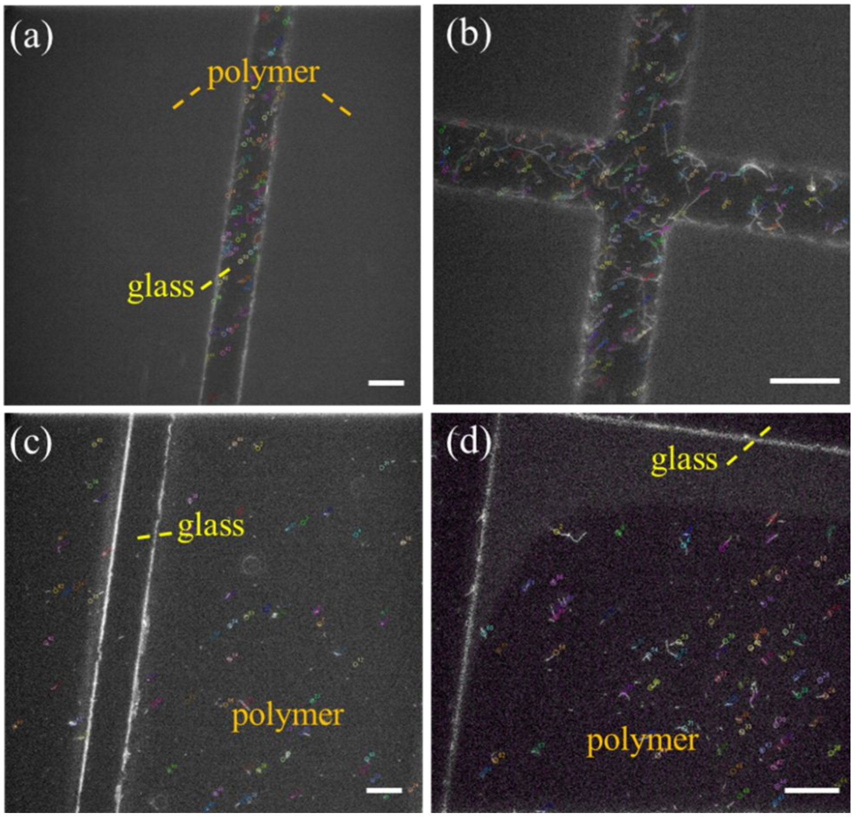

Fig. S1 Fluorescence images of actin filaments bound on microchannel floors (**a+b**) and on microchannel sidewalls (**c+d**)(scale bars correspond to 10 μm).

Toxicity and Handling hazards of the silane compounds:

	TMCS	FOTCS	FDDTCS
**Reactivity and Handling Hazards**			
Reactivity	Very reactive with water and alcohols	Also very reactive due to Si–Cl, hydrolyzes to form HCl	
Corrosive potential	High (generates HCl on contact with water)	Same, generates HCl	
Flammability	Flammable vapor	Less flammable (due to high fluorination)	
Volatility	Very volatile	Less volatile	Much less volatile
Inhalation risk	High due to volatility	Lower vapor pressure → lower inhalation risk	
**Toxicity**			
Acute Toxicity	Moderate to high (irritant, corrosive)	Not well-studied; expected low acute toxicity due to low absorption	
Skin/eye irritation	Severe	Moderate to severe (due to Si–Cl groups)	
Inhalation hazard	High	Lower (due to lower volatility)	Much (due to lower volatility)
Chronic exposure concern	Limited data	Potential concern from perfluoroalkyl chains	
**NFPA 704 hazard rating system**			
Health	3	2	2
Flammability	3	1	0-1
Reactivity	2	2	2
NFPA Code	3/3/2	2/1/2	2/0-1/2
**Pictograms**			
	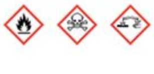	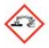	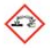

Fig. S2 Comparison of aspects important for safety considerations concerning the silane compounds (TMCS, FOTCS and FDDTCS) used in this study.

(a) FDDTCS (b) FOTCS (c) TMCS

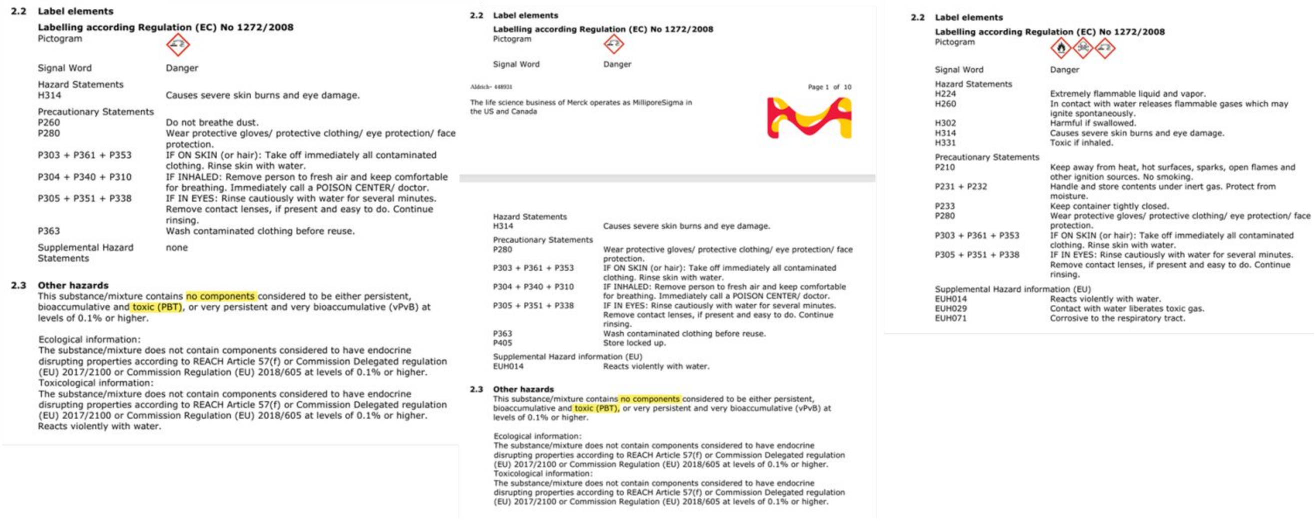

(a) FDDTCS

2.2 Label elements

Labelling according Regulation (EC) No 1272/2008

Pictogram	
Signal Word	Danger
Hazard Statements	
H314	Causes severe skin burns and eye damage.
Precautionary Statements	
P260	Do not breathe dust.
P280	Wear protective gloves/ protective clothing/ eye protection/ face protection.
P303 + P361 + P353	IF ON SKIN (or hair): Take off immediately all contaminated clothing. Rinse skin with water.
P304 + P340 + P310	IF INHALED: Remove person to fresh air and keep comfortable for breathing. Immediately call a POISON CENTER/ doctor.
P305 + P351 + P338	IF IN EYES: Rinse cautiously with water for several minutes. Remove contact lenses, if present and easy to do. Continue rinsing.
P363	Wash contaminated clothing before reuse.
Supplemental Hazard Statements	none

2.3 Other hazards

This substance/mixture contains no components considered to be either persistent, bioaccumulative and toxic (PBT), or very persistent and very bioaccumulative (vPvB) at levels of 0.1% or higher.

Ecological information:
The substance/mixture does not contain components considered to have endocrine disrupting properties according to REACH Article 57(f) or Commission Delegated regulation (EU) 2017/2100 or Commission Regulation (EU) 2018/605 at levels of 0.1% or higher.
Toxicological information:
The substance/mixture does not contain components considered to have endocrine disrupting properties according to REACH Article 57(f) or Commission Delegated regulation (EU) 2017/2100 or Commission Regulation (EU) 2018/605 at levels of 0.1% or higher.
Reacts violently with water.

(b) FOTCS

2.2 Label elements

Labelling according Regulation (EC) No 1272/2008

Pictogram	
Signal Word	Danger

Aldrich- 448931 Page 1 of 10

The life science business of Merck operates as MilliporeSigma in the US and Canada

Hazard Statements	
H314	Causes severe skin burns and eye damage.
Precautionary Statements	
P280	Wear protective gloves/ protective clothing/ eye protection/ face protection.
P303 + P361 + P353	IF ON SKIN (or hair): Take off immediately all contaminated clothing. Rinse skin with water.
P304 + P340 + P310	IF INHALED: Remove person to fresh air and keep comfortable for breathing. Immediately call a POISON CENTER/ doctor.
P305 + P351 + P338	IF IN EYES: Rinse cautiously with water for several minutes. Remove contact lenses, if present and easy to do. Continue rinsing.
P363	Wash contaminated clothing before reuse.
P405	Store locked up.
Supplemental Hazard information (EU)	
EUH014	Reacts violently with water.

2.3 Other hazards

This substance/mixture contains no components considered to be either persistent, bioaccumulative and toxic (PBT), or very persistent and very bioaccumulative (vPvB) at levels of 0.1% or higher.

Ecological information:
The substance/mixture does not contain components considered to have endocrine disrupting properties according to REACH Article 57(f) or Commission Delegated regulation (EU) 2017/2100 or Commission Regulation (EU) 2018/605 at levels of 0.1% or higher.
Toxicological information:
The substance/mixture does not contain components considered to have endocrine disrupting properties according to REACH Article 57(f) or Commission Delegated regulation (EU) 2017/2100 or Commission Regulation (EU) 2018/605 at levels of 0.1% or higher.

(c) TMCS

2.2 Label elements

Labelling according Regulation (EC) No 1272/2008

Pictogram	
Signal Word	Danger
Hazard Statements	
H224	Extremely flammable liquid and vapor.
H260	In contact with water releases flammable gases which may ignite spontaneously.
H302	Harmful if swallowed.
H314	Causes severe skin burns and eye damage.
H331	Toxic if inhaled.
Precautionary Statements	
P210	Keep away from heat, hot surfaces, sparks, open flames and other ignition sources. No smoking.
P231 + P232	Handle and store contents under inert gas. Protect from moisture.
P233	Keep container tightly closed.
P280	Wear protective gloves/ protective clothing/ eye protection/ face protection.
P303 + P361 + P353	IF ON SKIN (or hair): Take off immediately all contaminated clothing. Rinse skin with water.
P305 + P351 + P338	IF IN EYES: Rinse cautiously with water for several minutes. Remove contact lenses, if present and easy to do. Continue rinsing.
Supplemental Hazard information (EU)	
EUH014	Reacts violently with water.
EUH029	Contact with water liberates toxic gas.
EUH071	Corrosive to the respiratory tract.

Fig. S3 Screenshots from material safety data sheets (MSDS) of (a) FDDTCS, (b) FOTCS and (c) TMCS.

Reproducibility:

To demonstrate the reproducibility of our silanization protocol for samples used in IVMAs on flat Ormostamp polymer surfaces and glass slides, we repeated the experiments from the study several months apart. On both investigated materials (Ormostamp and glass slides) functionalized with FOTCS similar average sliding velocities and ratio of motile filaments were observed (**Fig. S4**), across repeated experiments.

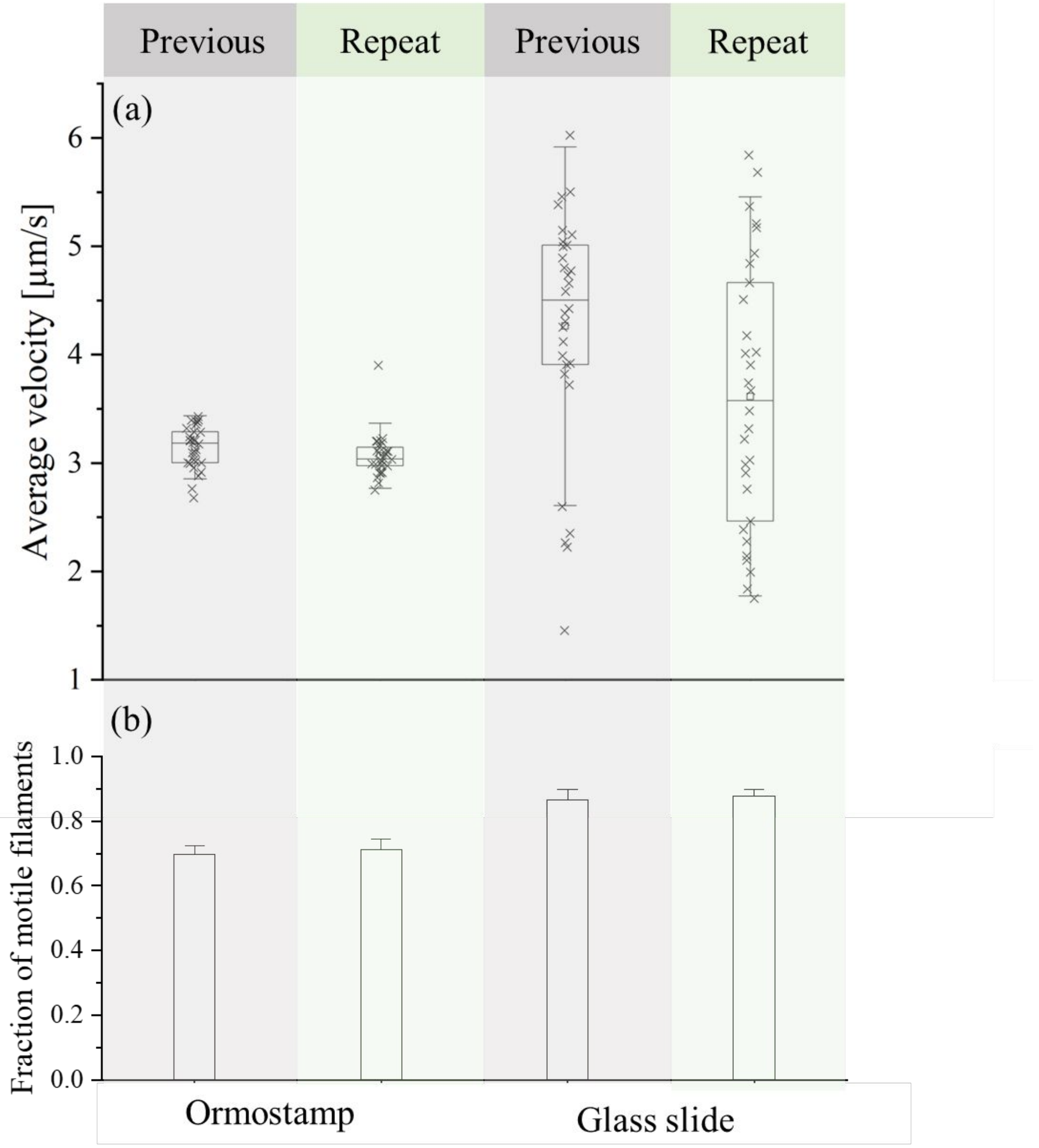

Fig. S4 (a) Box-plot of average sliding velocities of n = 30 individual actin filaments on flat Ormostamp polymer surfaces and on glass slides in comparison with repeated experiments, where

the box gives the middle two quartiles of the data, the central line represents the median. with FOTCS. (b) Fraction of motile filaments measured on flat Ormostamp polymer surfaces and on glass surfaces in comparison with repeated experiments. Error bars in (b) represent the standard deviation from the mean of three individual samples. IVMA performed at 22°C.

Statistical significance tests:

We have run statistical significance tests on the results on the motility assays (see results on Table S2). We observe that the difference in the speeds is statistically significant (ANOVA: $F(2,117)=19$, $p=7\cdot10^{-8}$), with large size effect, also when comparing the samples one to one. When the IVMA were repeated using three different samples prepared under similar conditions and measured within a few days, no statistically significant variability was observed ($F(2,27)=0.001$, $p=0.999$). We also observed that repeating the measurements several months later using FOTCS to coat Ormostamp and performing the IVMA with different motors and filament batches also resulted in no significant variability ($F(1,58)=2.3$, $p=0.13$. The same type of repetition using FOTCS to coat glass resulted, on the other hand, in statistically significant differences ($F(1,58)=4.6$, $p=0.036$). However, the effect size is small (Cohen's $f=0.28$), suggesting that although the differences are statistically significant, the practical impact is limited.

Tab. S2 Summary of ANOVA statistical significance tests performed on IVMA data sets used in this study. The results are classified as significant or not significant (n.s.) based on the p-value (p<0.05, Statistically Significant; p>0.05, Not Significant). Effect size interpretation: cohen's f < 0.10, Negligible; 0.10 – 0.25, Small to Moderate; 0.25 – 0.40, Moderate to Large; > 0.40, Large

Comparison	F_{crit}	F-value	p-value	Significance	df (between,within)	η²	Cohen's f	Effect size
**Different silanes on glass**								
TMCS, FOTCS, FDDTCS	3.07	19	$7*10^{-8}$	Significant	(2,117)	0.244	0.57	Large
TMCS, FOTCS	3.95	12	0.00079	Significant	(1, 88)	0.12	0.369	Moderate–Large
TMCS, FDDTCS	4	8	0.0063	Significant	(1, 58)	0.121	0.371	Moderate–Large
FOTCS, FDDTCS	3.95	27	<0.001	Significant	(1, 88)	0.235	0.554	Large
**Repetitions**								
FOTCS on glass: three repeats, same day	3.3	0.001	0.999	N.S.	(2, 27)	$7*10^{-5}$	0.009	Negligible
FOTCS on glass: previous, repeat	4	4.6	0.036	Significant	(1, 58)	0.074	0.281	Small–Moderate
FOTCS on Ormo: previous, repeat	4	2.3	0.13	N.S.	(1, 58)	0.038	0.2	Small-Moderate

Files:

Tab. S3 List of submitted files. Video files V1-V5 were acquired during IVMAs at different conditions and used to determine average filament velocities. All IVMAs were performed at 22°C. All other files are the submitted MSDS of the silane compounds used in this study.

File name	Content/Condition
V2_ FOTCS on glass	FOTCS on glass
V3_ FDDTCS on glass	FDDTCS on glass
V4_ FOTCS on ormostamp	FOTCS on ormostamp
V5_ FOTCS on glass with traced filaments	FOTCS on glass with traced filaments
V6_ WCA measurement @2.5 minutes of UV-Ozone treatment	WCA measurement @2.5 minutes of UV-Ozone treatment
TMCS_MSDS	Safety information for Trimethylchlorosilan
FOTCS_MSDS	Safety information for Perfluoro-octyltrichlorosilane
FDDTCS_MSDS	Safety information for Perfluoro-dodecyltriochlorosilane

Tables for Filament velocities and WCA:

The following tables list the filament velocities and WCA used for the plots in **Fig. 3**, **Fig. 5**, **Fig. 7** and **Fig. 8**.

Tab. S4 List of WCA plotted in **Fig. 3** and filament velocities used for plots in **Fig. 5**, **Fig. 7** and **Fig. 8**.

Condition	WCA [°]
FOTCS, 400 mbar, 85°C (Fig. 3(c))	88,5
	89,9
	91,1
	90,2

90,6

90

89,7

89,9

90

91

87

86

88

89,5

89,4

91

90

89,6

90,9

89

90

88,4

89,2

90,1

88,9

89

87,6

90,3

89,5

88,8

90

89,1

88,6

87,9

89,8

88,3

90,2

89,7

88,7

89,6

87,8

88,9

89

90,4

90,1

90,4

89,8

90,2

90,5

89,9

90

90,6

90,3

91

89,7

90,1

90,2

91,1

90,7

89,6

90,4

90,2

91,2

89,5

90,3

90

90,5

91

89,8

90,1

91,3

90,6

89,9

90,2

89,7

91,4

90,3

89,6

91

89,4

90,8

90,5

90,1

90,2

89,9

91,1

90,6

89,8

	90,4
	89,5
	91,2
	88,5
	Filament velocity [µm/s]

Condition	Filament velocity [µm/s]
FOTCS coating, glass, ambient temperature (Fig. 5)	5,384
	4,425
	3,909
	5,459
	5,502
	3,72
	5,04
	6,024
	5,146
	2,353
	2,264
	2,597
	1,454
	2,223
	4,255
	3,921
	4,658
	4,892
	5,01
	4,998

4,305
4,382
5,106
4,8
4,772
3,82
4,119
4,732
4,582
3,989
3,482
5,173
2,986
4,841
3,221
1,993
2,465
4,012
5,369
2,758
3,904
4,177
1,839
2,144
3,028
4,665
5,21
2,387

	3,74
	4,935
	2,104
	1,752
	5,682
	2,908
	4,024
	3,316
	2,276
	4,509
	3,667
	5,841
FDDTCS coating, glass, ambient temperature (**Fig. 5**)	1,314
	1,091
	3,426
	2,386
	2,218
	2,066
	4,234
	1,159
	1,061
	2,961
	2,056
	1,681
	1,929
	1,4
	2,603

	3,71
	3,554
	1,307
	3,9
	3,375
	3,002
	3,204
	3,339
	2,577
	3,503
	3,694
	3,001
	2,875
	2,78
	3,264
TMCS coating, glass, ambient temperature (**Fig. 5**)	3,221
	3,393
	3,813
	3,148
	2,989
	3,917
	3,199
	2,128
	2,146
	3,149
	2,976
	3,411
	3,607

	3,1
	2,974
	3,005
	3,164
	2,7
	3,554
	3,088
	2,732
	2,998
	3,25
	3,02
	2,799
	3,408
	3,21
	3,093
	3,87
	3,442
FOTCS coating, polymer, ambient temperature (Fig. 7)	2,995
	3,188
	3,393
	2,916
	3,003
	3,356
	3,287
	3,241
	3,212
	2,88
	3,21

3,319

3,378

3,398

2,761

3,098

3,123

3,014

3,429

3,062

3,027

3,388

3,175

3,003

3,215

3,203

2,68

3,097

3,285

2,955

3,098

3,208

3,102

2,99

2,91

2,75

3,2

3,031

3,2

2,905

	3,015
	3,145
	2,975
	3,08
	2,92
	3,19
	2,865
	3,11
	3,002
	3,225
	3,902
	3,032
	2,816
	2,975
	3,107
	2,993
	3,042
	3,17
	2,913
	3,107
FOTCS coating, polymer microchannels, ambient temperature (Fig. 8)	3,134
	2,423
	2,781
	2,908
	2,477
	3,341
	2,757

3,325
2,674
3,154
2,976
2,1
2,765
2,693
2,648
3,264
3,329
2,633
2,672
3,172
2,635
3,004
3,08
3,007
2,956
3,132
3,316
2,995
3,01
2,774
